# Dual Roles of m^6^A Modification: Orchestrating Development and Abiotic Stress Resilience in Plants

**DOI:** 10.3390/cells15100943

**Published:** 2026-05-20

**Authors:** Yang Sun, Wen Qin, Yiting Gong, Yinqiao Jian, Fangling Jiang, Rosa M. Rivero, Ron Mittler, Zhen Wu, Rong Zhou

**Affiliations:** 1 Sanya Research Institute of Nanjing Agricultural University, College of Horticulture, Nanjing Agricultural University, Nanjing 210095, China; 2024104071@stu.njau.edu.cn (Y.S.); qinwen@stu.njau.edu.cn (W.Q.); 2025104070@stu.njau.edu.cn (Y.G.); jfl@njau.edu.cn (F.J.); zpzx@njau.edu.cn (Z.W.); 2 Key Laboratory of Biology and Genetic Improvement of Tuber and Root Crop of Ministry of Agriculture and Rural Affairs, Institute of Vegetables and Flowers, Chinese Academy of Agricultural Sciences, Beijing 100081, China; jianyinqiao@caas.cn; 3 Department of Plant Nutrition, Center of Edaphology and Applied Biology of Segura (CEBAS-CSIC), Campus Universitario Espinardo, Ed 25, 30100 Murcia, Spain; rmrivero@cebas.csic.es; 4 Division of Plant Science and Technology, College of Agriculture, Food and Natural Resources, Bond Life Sciences Center, University of Missouri, Columbia, MO 65201, USA; mittlerr@missouri.edu; 5 Advanced Agriculture Initiative, Tel-Hai University of Kiryat Shmona and the Galilee, Kiryat Shmona 1220800, Israel

**Keywords:** RNA N^6^-methyladenosine, plant development, abiotic stress response, mRNA stability, mRNA translation efficiency, alternative polyadenylation, histone modification

## Abstract

**Highlights:**

**What are the main findings?**

- Writers, readers, and erasers control mRNA fate, thereby orchestrating plant development.
- m^6^A regulators mediate tolerance to abiotic stresses with pronounced species, cultivar, and tissue specificity.

**What are the implications of the main findings?**

- By systematically organizing current knowledge on m^6^A regulators and their mechanisms in both development and stress responses, this review serves as a valuable resource for researchers in related fields and identifies critical gaps for future investigation.
- The observed species and cultivar specificity of m^6^A patterns, combined with emerging understanding of m^6^A regulatory mechanisms, lays a theoretical foundation for potentially engineering RNA modifications to enhance stress resilience or optimize developmental traits in crops.

**Abstract:**

RNA N^6^-methyladenosine (m^6^A) is a prevalent epitranscriptomic modification that governs plant growth, development, and environmental adaptation. This review synthesizes recent advances in understanding the molecular mechanisms and biological functions of m^6^A in plants. The m^6^A landscape is dynamically regulated by methyltransferases (writers), demethylases (erasers), and m^6^A-binding proteins (readers), which collectively influence mRNA stability, translation efficiency, alternative polyadenylation (APA), and chromatin crosstalk. Functionally, m^6^A integrates diverse developmental processes—including embryogenesis, organogenesis, flowering, fruit ripening, and leaf senescence—with abiotic stress responses such as salt, drought, cold, and heat. Notably, m^6^A modification exhibits remarkable species-, cultivar-, and tissue-specific plasticity, enabling precise spatiotemporal gene regulation. Recent breakthroughs have revealed bidirectional crosstalk between m^6^A and histone modifications, forming a multi-layered regulatory network, while emerging concepts including phase separation, RNA structure dynamics, and stress memory further expand the functional repertoire of m^6^A. Despite significant progress, plant epitranscriptomics remains mechanistically underexplored, with critical gaps persisting in our understanding of translation initiation mechanisms, upstream regulatory signals controlling writers/erasers activities, and the functional significance of individual m^6^A sites. This review provided systematic insights into the complexity and specificity of m^6^A regulation in plants, offering a theoretical foundation for future efforts to decipher and ultimately manipulate this epitranscriptional layer for crop improvement.

## 1. Introduction

**Figure 1:**
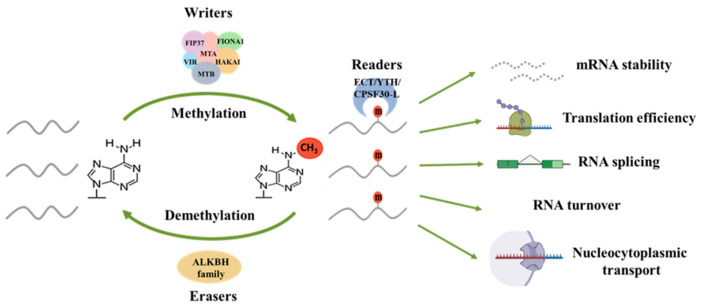
m^6^A-mediated regulation of mRNA fate. m^6^A writers (methyltransferases) complex catalyze the addition of m^6^A modifications; readers (m^6^A-binding proteins) recognize m^6^A marks and mediate downstream functions; erasers (demethylases) remove m^6^A modifications. m^6^A modification controls multiple aspects of mRNA metabolism, including RNA stability, translation efficiency, RNA splicing, RNA turnover and nucleocytoplasmic transport. Through distinct reader proteins, m^6^A determines the post-transcriptional fate of target mRNAs.

RNA modification, a crucial post-transcriptional regulatory mechanism, constitutes an important layer of epigenetics. Studies have identified over a hundred types of RNA modifications across eukaryotic mRNA, tRNA, rRNA, small non-coding RNA (snRNA), microRNA (miRNA), and long non-coding RNA (lncRNA) [1,2,3]. Among these, the addition of N^6^-methyladenosine (m^6^A) to mRNA is one of the most widespread and intensively researched types [4]. The m^6^A is not uniformly distributed across transcripts but is predominantly enriched near stop codons and within 3′ untranslated regions (3′ UTR) [5]. Transcriptome-wide mapping further reveals that m^6^A typically occurs within the conserved RRACH motif (R = A/G, H = A/C/U) [6,7]. Plants also possess a unique motif, URUAY (R = G > A, Y = U > A), located at the mRNA 3′ UTR [8]. Genetic and transcriptomic studies collectively indicated that m^6^A plays key roles in enhancing mRNA stability [9,10,11], promoting translation efficiency [12,13,14], as well as influencing splicing [15,16], RNA turnover, and nucleocytoplasmic transport [17] (Figure 1). These findings underscore the central role of co-transcriptional and post-transcriptional RNA chemical modifications in determining mRNA fate (Figure 1). Despite rapid advances in identifying m^6^A regulators in plants, our understanding of how this modification integrates developmental programs with abiotic stress responses remains incomplete. In this review, we discuss the molecular machinery controlling m^6^A dynamics, examine its roles in plant development and abiotic stress adaptation, and highlight emerging concepts and future directions in plant epitranscriptomics.

## 2. m^6^A Writers, Readers, and Erasers: A Tripartite Synergistic System for Plant Epitranscriptomic Regulation

As a dynamic and reversible post-transcriptional marker [18], the addition or removal of m^6^A to nuclear mRNAs are mediated by methyltransferases (writers) or demethylases (erasers), respectively, while m^6^A-binding proteins (readers) recognize and bind modified sites to exert regulatory functions [19,20,21] (Figure 1).

Writers add m^6^A modifications to mRNA after recognizing specific motifs as substrates [6,7,8]. Writers are categorized into two types. The first includes multi-protein complexes composed of MTA (homolog of human METTL3), MTB (homolog of human METTL14), FIP37 (homolog of human WTAP), VIRILIZER (VIR) (homolog of human VIRMA), HAKAI (a ubiquitin E3 ligase), and HIZ2 (HAKAI-interacting zinc finger protein) (homolog of human ZC3H13). The second is the recently discovered single-protein writer FIONA1 [22,23,24,25]. Multiple studies have characterized that m^6^A writers play crucial roles in both plant development and abiotic stress responses. In terms of plant development, these factors are widely involved in various biological processes ranging from embryonic development and stem-cell differentiation to fruit ripening and floral transition [22,24,26,27,28,29]. In terms of abiotic stress responses, they are primarily involved in drought, low-temperature, and salt stresses, influencing plant tolerance by regulating mechanisms such as lignin synthesis, reactive oxygen species (ROS) scavenging, and mRNA stability [14,30,31].

Readers selectively recognize m^6^A signals added by writers, influencing the subsequent fate of transcripts [32]. Currently known readers mainly include YTH (YT521-B homology) domain proteins and KH (K-homology) domain proteins [33]. The most extensively studied YTH domain proteins rely on their conserved YTH domain for recognition and binding, which contains a hydrophobic pocket formed by four α-helices and six β-strands [34,35]. In total, 13 YTH domain proteins have been identified in *Arabidopsis* [36], with 11 belonging to the ECT (EVOLUTIONARILY CONSERVED C-TERMINAL REGION) family, including ECT1 to ECT12, and CPSF30-L (CLEAVAGE AND POLYADENYLATION SPECIFICITY FACTOR 30), all recognized as YTH family members [37,38,39].

Erasers can remove m^6^A modifications already formed on coding RNAs. This reversibility ensures that the methylation level within plants is a dynamically changing process under strict spatiotemporal regulation, serving as an important molecular basis for plants to adapt to complex and variable natural environments [32,40]. Currently, several members of the ALKBH (the a-ketoglutarate-dependent dioxygenase homolog) family have been identified as plant erasers [41], playing significant roles in various stages of growth and development, abiotic stress responses, and viral infection [28,42,43].

## 3. Mechanisms by Which m^6^A Controls Gene Expression

### 3.1. m^6^A and mRNA Stability

The relationship between m^6^A and mRNA stability is complex. In apple MdMTA RNA interference (RNAi) plants, reduced methylation levels correlate with decreased stability of genes involved in lignin deposition and oxidative stress response [44]. Anderson et al. [45] found that m^6^A can be dynamically deposited in salt stress response-related genes, enhancing their mRNA stability. However, m^6^A regulation of transcript stability is not always positive. m^6^A modification on shoot apical meristem (SAM) gene mRNAs restricts their transcript abundance, preventing meristem over-proliferation [27]. Similarly, mutation in the eraser *slalkbh2* negatively affects DNA demethylase *SlDML2* mRNA stability, delaying tomato fruit ripening [28]. These results indicate that the effects of m^6^A on stability are highly context-dependent. While an increasing number of studies point to the relationship between m^6^A and mRNA stability, the underlying molecular mechanisms remain poorly understood. Hu et al. [46] revealed a direct mechanism whereby m^6^A modification itself can physically protect transcripts from degradation. In this model, the presence of the methyl group at specific adenosine residues creates a steric hindrance that prevents recognition and cleavage by endonucleases. Additionally, m^6^A can function by altering mRNA secondary structure [9]. Kramer et al. [9] elegantly linked epitranscriptomic marks with RNA structural dynamics, demonstrating that the methyl group induces a looser secondary structure in the mRNA of the salt stress-responsive gene *P5CS1* (*DELTA 1-PYRROLINE-5-CARBOXYLATE SYNTHASE*). This structural relaxation enhances accessibility for stabilizing RNA-binding proteins while potentially occluding recognition sites for degradation factors, ultimately leading to increased mRNA stability [9]. Cai Z et al. [47] also reported that ECT8 mediates the 5′-end de-capping degradation pathway. Specifically, ECT8 recognizes the m^6^A mark and recruits DCP5 (DECAPPING 5), a key component of the de-capping complex, to the 5′ end of target genes, initiating the 5′-to-3′ mRNA degradation pathway for negative stress regulators to enable an effective response to abiotic stress.

In summary, while m^6^A is known to either stabilize or destabilize mRNAs depending on context through mechanisms including steric hindrance, RNA structural remodeling, or reader-mediated degradation [9,27,28,44,45,46,47], it remains unclear what determines these opposing outcomes and how different readers are coordinated on the same transcript.

### 3.2. Regulation of Translation Efficiency

#### 3.2.1. Evidence for m^6^A-Promoted Translation Efficiency

In the *Arabidopsis mta* knockdown mutant *AmiR-mta*, the mRNA abundance of *DGAT1* (*DIACYLGLYCEROL ACYLTRANSFERASE 1*) did not significantly decrease compared to wild-type under both normal and cold conditions, yet its translation efficiency was negatively affected, leading to reduced protein expression [14]. This finding is critically important because it definitively demonstrates that m^6^A can directly regulate translation efficiency through mechanisms independent of stability, without altering transcript abundance. Similarly, Govindan et al. [48] employed polysome profiling to demonstrate that transcripts with high m^6^A levels exhibit higher ribosome occupancy, further supporting the positive role of m^6^A in translation efficiency. The strawberry writer MTA regulates strawberry fruit ripening by targeting the translation efficiency of genes in the abscisic acid (ABA) signaling pathway [13]. Under drought conditions, apple MdMTA effectively promotes the translation efficiency of genes involved in lignin biosynthesis and oxidative stress responses [44]. m^6^A-mediated translational control thus emerges as a conserved regulatory mechanism across species in plant development and stress adaptation.

#### 3.2.2. Eukaryotic Initiation Factor 3 Recruitment Mechanism

In animal systems, m^6^A modification within the 5′ UTR can directly bind the eukaryotic translation initiation factor eukaryotic initiation factor 3 (eIF3), bypassing the cap-binding protein eukaryotic initiation factor 4E (eIF4E) and directly recruiting the 43S pre-initiation complex, thereby initiating cap-independent translation [49]. In plants, emerging evidence supports a similar mechanism. For instance, in rice, the OseIF3h physically interacts with OsMTA2, as demonstrated by yeast two-hybrid and co-immunoprecipitation assays [50]. Loss of OseIF3h function leads to severe growth retardation, reduced seed-setting rate, and abnormal pollen development, accompanied by attenuated translation efficiency of specific gene subsets [50]. These findings indicate that the functional coupling between m^6^A modification and eIF3-mediated translation initiation is at least partially conserved in plants, laying the groundwork for future investigations into cap-independent translation mechanisms [49,50].

#### 3.2.3. Coordination with RNA-Binding Proteins

In rice, the RNA-binding protein EHD6 directly interacts with the m^6^A reader YTH07 [51]. Notably, EHD6 not only physically associates with YTH07 but also promotes its m^6^A-binding activity and facilitates its phase separation into ribonucleoprotein condensates [51]. These condensates sequester target mRNAs and repress their translation, thereby regulating flowering time [51]. This study provides a striking example of how an RNA-binding protein can modulate the function of an m^6^A reader to achieve specific translational control.

#### 3.2.4. Translational Reprogramming Under Stress

Under stress conditions, global translation is suppressed to conserve energy, while translation of key stress-responsive genes is selectively activated [52]. m^6^A modification orchestrates this translational reprogramming. Under cold stress, m^6^A ensures preferential translation of cold-responsive transcripts while suppressing growth-related genes [14]. This selective activation enables rapid proteome remodeling, prioritizing survival over growth [14].

Thus, m^6^A is known to promote translation efficiency in multiple plant species independently of mRNA stability and to orchestrate translational reprogramming under stress, with recent evidence from rice showing physical interaction between eIF3 and the m^6^A writer complex [13,14,44]. However, whether plant readers directly recruit eIF3, whether cap-independent translation exists in plants, and how multiple readers coordinate translation control remain open questions.

### 3.3. m^6^A Participates in Alternative Polyadenylation

Alternative polyadenylation (APA) generates mRNA isoforms with distinct 3′ UTR lengths by selecting among different poly(A) sites [53,54], thereby influencing mRNA stability, translation efficiency, protein subcellular localization, and other biological processes through post-transcriptional regulation [55,56]. Recent studies have revealed extensive crosstalk between m^6^A modification and APA processes.

In the *Arabidopsis cpsf30-L* knockout mutant, more than half of all genes exhibit altered poly(A) site selection, with a pronounced shift toward proximal poly(A) sites [38]. This finding demonstrates that CPSF30-L, as an m^6^A reader, participates in regulating APA site choice through recognition of m^6^A modifications [38]. Under salt stress, VIR-mediated m^6^A marks influence APA site selection, preventing 3′ UTR lengthening of stress-related genes and thereby promoting transcript degradation [46]. Govindan et al. [48] further revealed that m^6^A facilitates the production of mRNA isoforms with shorter 3′ UTRs for cold-responsive genes, potentially allowing these transcripts to escape recognition by cleavage factors and enhancing their stability and translation efficiency.

Collectively, the m^6^A reader CPSF30-L is known to regulate poly(A) site choice, VIR-mediated m^6^A prevents 3′ UTR lengthening of stress-related genes, and cold stress induces m^6^A-facilitated production of shorter 3′ UTR isoforms [38,46,48]. Nevertheless, how m^6^A physically influences the polyadenylation machinery and what determines whether it promotes proximal versus distal site usage remain to be elucidated.

## 4. Crosstalk with Chromatin and Other Epigenetic Layers: Bidirectional Regulation and Transcriptional Coupling

### 4.1. Histone Modifications Guide Co-Transcriptional m^6^A Deposition

Events occurring within the nucleus directly influence the subsequent fate of RNA products. Chromatin states, particularly specific histone modifications, can serve as upstream signals that guide m^6^A deposition on nascent RNA chains, achieving coupling between transcriptional and post-transcriptional regulation [57,58]. In animal systems, this mechanism has been clearly elucidated: histone H3 lysine 36 trimethylation (H3K36me3)—a mark associated with active transcriptional elongation-recruits the writers complex component METTL14, thereby co-transcriptionally adding m^6^A modifications to nascent RNAs [57]. This coupling ensures spatiotemporal coordination between m^6^A modification and gene transcriptional activity. Emerging evidence suggests that similar conserved mechanisms may operate in plants. For instance, Shim et al. [58] demonstrated that H3 lysine 36 dimethylation (H3K36me2) levels in *Arabidopsis* strongly correlate with m^6^A abundance on target gene mRNAs, raising the possibility that H3K36me2 could function as an upstream signal influencing m^6^A deposition. It is important to note, however, that this remains speculative at present, as the direct molecular bridge connecting histone modifications to m^6^A writers in plants has not yet been identified. Nevertheless, this correlation suggests a hypothesis worth testing: that chromatin-state-guided RNA methylation might represent a conserved regulatory mechanism.

### 4.2. m^6^A Reciprocally Regulates Chromatin State and Transcription

More revolutionary is the discovery that this crosstalk is not unidirectional but bidirectional: m^6^A modifications themselves can reciprocally influence the chromatin state from which they originated, forming a closed-loop regulatory system [59]. Song et al. [59] achieved a breakthrough in *Arabidopsis*: they discovered that m^6^A modifications on retrotransposon RNAs can be recognized by specific reader proteins. Subsequently, these readers recruit histone methyltransferases to corresponding genomic DNA loci, catalyzing the addition of repressive Histone H3 lysine 9 dimethylation (H3K9me2), thereby compacting chromatin and suppressing retrotransposon transcription [59].

### 4.3. An Integrated Perspective: A Coupled Transcriptional–Epitranscriptional Regulatory Circuit

Taken together, an integrated model emerges: during transcriptional elongation, active chromatin states recruit writer complexes, guiding co-transcriptional m^6^A deposition on nascent RNAs [57,58]. These m^6^A marks subsequently influence RNA stability, splicing, or translation efficiency through reader proteins [57,58]. More critically, at certain loci such as transposons, these RNA m^6^A marks can, via specific readers, reciprocally recruit chromatin-modifying complexes to reshape the local chromatin environment, thereby influencing future transcriptional initiation [59]. This bidirectional coupling ensures the precision and robustness of gene expression regulation, enabling cells to coordinately fine-tune gene output at both transcriptional and post-transcriptional levels in response to different developmental stages and environmental signals [57,58,59]. This emerging research field not only challenges traditional disciplinary boundaries but also provides novel perspectives for understanding complex life phenomena. Future research should prioritize exploring: What are the specific molecular mechanisms linking histone modifications to m^6^A writers in plants? Which readers mediate the reverse signal from m^6^A to chromatin modifications? How is this crosstalk dynamically regulated during plant development and environmental responses?

## 5. Emerging Concepts in Plant m^6^A Biology

### 5.1. Single-Cell Heterogeneity of m^6^A

Methylated RNA immunoprecipitation sequencing (MeRIP-seq) is based on bulk tissue samples, and the resulting methylation profiles represent average signals across different cell types, masking intercellular heterogeneity [6]. Single-cell m^6^A sequencing (scm^6^A-seq) enables the acquisition of genetic information at single-cell resolution, revealing the cell type-specific distribution of m^6^A modifications [60]. In animal systems, studies have demonstrated the potential regulatory functions of m^6^A regulatory factors across different cell types [61,62]. In plants, however, scm^6^A-seq technology has not yet been maturely applied. In the future, integrating scm^6^A-seq with multi-omics analyses such as single-cell transcriptomics and single-cell ATAC-seq will comprehensively reveal the regulatory mechanisms of m^6^A modification in cell fate determination.

### 5.2. Phase Separation and m^6^A-Mediated Condensate Formation

m^6^A-modified RNAs and m^6^A readers can undergo liquid-liquid phase separation, forming membraneless organelles that concentrate specific transcripts and regulatory factors [63]. m^6^A modification and its readers have been established as key components of phase separation, orchestrating spatiotemporally specific regulation of target transcripts through recruitment of translation machinery or degradation factors [64,65]. Multiple m^6^A readers such as ECT1, ECT2, CPSF30-L, SiYTH1, and SlYTH2 participate in mRNA metabolism during heat stress, drought stress, salt stress, and immune responses by forming membraneless biomolecular condensates [39,66,67,68,69]. Furthermore, ALKBH9B-mediated demethylation influenced condensate formation and disassembly, thereby affecting the subcellular localization and fate of transcripts [70]. Phase separation therefore functions as a core mechanism of m^6^A-mediated RNA metabolism, with critical implications for plant development and stress adaptation.

### 5.3. Stress Memory and Transgenerational Inheritance

Whether stress-induced m^6^A patterns persist after stress relief and contribute to stress memory, which is defined as enhanced responses upon subsequent stress encounters, remains unknown. Even more provocatively, whether m^6^A modifications can be transmitted across generations, contributing to transgenerational stress adaptation, represents a fundamental question with profound implications for understanding plant environmental responses and evolution.

## 6. Epitranscriptomic Plasticity: Species, Cultivar, and Tissue Specificity

### 6.1. Species-Specific m^6^A Landscapes

In response to the same type of stress, m^6^A response patterns are not uniform across all plants and exhibit high complexity and species specificity [48,71,72]. In *Arabidopsis*, cold stress induces a significant increase in transcriptome-wide m^6^A levels [48]. This global increase is considered a conserved stress response, potentially suppressing growth-related genes by generally reducing transcript stability while reallocating resources to activate cold adaptation pathways [48]. In contrast, cold stress leads to a genome-wide decrease in m^6^A levels in tomato anthers [71]. This trend may be related to a protective mechanism unique to reproductive organs, which involves global demethylation to maintain mRNA stability of genes critical for fertility, prioritizing reproductive development under stress [71]. Mango seedlings exhibit another unique adaptation mechanism: cold stress-induced m^6^A modification enhances the binding of the molecular chaperone HSP70 (HEAT SHOCK PROTEIN 70) to specific methylated RNAs [72]. This does not directly indicate a simple global increase or decrease but reflects the functional specificity of m^6^A-mediated RNA-protein interactions at the translational regulation level, enhancing tolerance by promoting correct folding of cold-responsive proteins [72].

### 6.2. Cultivar-Specific Methylation Patterns

Different cultivars of the same species also show significant differences in m^6^A modification under stress, and these differences are closely related to resistance phenotypes [73,74,75].

#### 6.2.1. Cultivar-Specific m^6^A Modifications Exert Bidirectional Regulation on Stress-Responsive Genes

Ma L et al. [73] analyzed whole-transcriptome m^6^A profiles and differential gene expression profiles in *Brassica rapa* varieties with different cold tolerance under low temperature. While both the cold-tolerant cultivar L7 and the cold-susceptible cultivar Lenox showed decreased methylation levels, the decrease was more pronounced in L7. Concurrently, m^6^A enrichment at the middle region of the *ZAT12* (*ZINC FINGER OF ARABIDOPSIS THALIANA 12*) gene increased significantly in L7, but not in Lenox, indicating that m^6^A positively regulated *ZAT12* expression to enhance cold tolerance [73]. Conversely, m^6^A levels of the *MYBC1* (*MYB DOMAIN PROTEIN C 1*) gene increased significantly in Lenox but not in L7, suggesting that m^6^A negatively regulated *MYBC1* expression, suppressing cold tolerance in *Brassica rapa* [73]. Remarkably, m^6^A modifications can steer the same transcript toward opposite regulatory fates in different cultivars, enabling genotype-specific stress adaptation strategies.

#### 6.2.2. Cultivar-Specific Differences in m^6^A Modifications Affect the Activation Efficiency of Stress Signaling Pathways

Under mild drought treatment, the drought-resistant cotton cultivar ZY168 and the drought-sensitive cotton cultivar ZY7 exhibited significant differences in m^6^A levels under mild drought treatment, with ZY168 accumulating more m^6^A modifications in the 5′ untranslated regions (5′ UTR) [74]. These differential methylation patterns enabled the drought-resistant cultivar to regulate the abundance of transcripts associated with Ca^2+^ and ABA signaling pathways via m^6^A marks, generating a faster and stronger stress response [74]. In potato salt stress response, different cultivars displayed differential response patterns to StALKBH10B-mediated m^6^A demethylation [75]. StALKBH10B negatively regulates potato salt tolerance by removing m^6^A modifications from transcripts involved in flavonoid metabolism and ABA signaling pathways, and the efficiency of this regulatory mechanism varies among cultivars, directly contributing to differences in salt tolerance [75]. Cultivar-specific m^6^A differences thus manifest not merely as global methylation shifts, but as fine-tuned control over the activation efficiency of specific signaling pathways [74,75].

### 6.3. Tissue- and Stage-Specific Regulation

m^6^A modification also displays differential distribution and function across different tissues or developmental stages within the same plant [28,43,71,76]. Wang L et al. [43] found that *Arabidopsis* floral organs exhibited higher transcriptome-wide levels of m^6^A than leaves under heat stress, with distinct modification patterns: modifications were more enriched in coding sequence (CDS) regions in flowers, while more located in the 3′ UTR in leaves. More pronounced methylation in flowers negatively regulated gene expression variability, providing a new perspective for understanding why floral organs are more sensitive to high-temperature stress during heat waves [43,76]. Zhou et al. [28] monitored transcriptome-wide methylation levels at different ripening stages of tomato fruit, revealing a dynamic decreasing trend in m^6^A levels. Yang et al. [71] found that moderate low-temperature (MLT) treatments disrupt the m^6^A modification pattern of anther development-related genes in anthers, preventing normal gene expression, causing metabolic disorders, and ultimately leading to pollen abortion due to excessive ROS accumulation. m^6^A does more than simply respond to environmental stress signals—it interprets and executes them according to cell type and developmental state. This context-dependent logic enables precise post-transcriptional regulation that coordinates growth, development, and stress adaptation [28,43,71,76].

## 7. m^6^A Regulation of Plant Development

Multiple m^6^A regulatory factors have been functionally characterized in plant developmental processes (Table 1). Writers such as MTA, MTB, and FIP37 regulate embryonic development, stem cell maintenance, and fruit ripening across *Arabidopsis*, strawberry, and rice. Readers including ECT family members and CPSF30-L control leaf morphology, seed germination, and floral transition, while erasers such as ALKBH2, ALKBH5 and ALKBH10B participate in fruit ripening, flowering, and seed germination. Collectively, these findings establish the m^6^A machinery as a central regulator of plant development from embryogenesis to reproduction (Table 1).

### 7.1. Embryogenesis and Early Development

**Figure 2:**
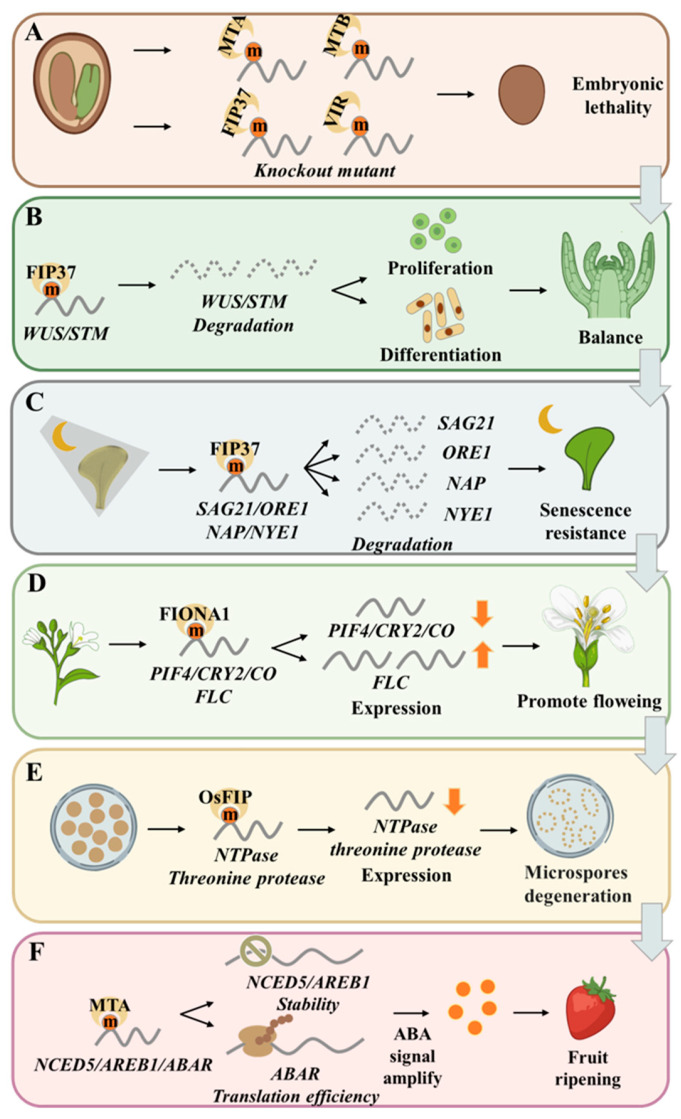
The multifaceted roles of m^6^A RNA methylation in plant development. (**A**) Knockout mutants of core components of the m^6^A writer complex (MTA/homolog of human METTL3, MTB /homolog of human METTL14, FIP37/homolog of human WTAP, VIR/homolog of human VIRMA) lead to embryonic lethality, indicating that m^6^A is essential for early embryogenesis in plants. (**B**) FIP37-mediated m^6^A modification promotes the degradation of transcripts of key developmental genes, *WUS* (*WUSCHEL*) and *STM* (*SHOOTMERISTEMLESS*), thereby balancing cell proliferation and differentiation to maintain the function of the SAM (shoot apical meristem). (**C**) FIP37 promotes the degradation of senescence-associated gene transcripts such as *SAG21* (*SENESCENCE-ASSOCIATED GENE 21*), *ORE1* (*ORESARA 1*), *NAP* (*NAC-LIKE, ACTIVATED by AP3/PI*), and *NYE1* (*NON-YELLOWING 1*) through m^6^A modification, thereby delaying the leaf senescence process and enhancing the plant’s anti-aging capacity. (**D**) FIONA1 promotes flowering in plants by regulating the m^6^A levels and expression abundance of flowering- and light sensing- related genes such as *PIF4* (*PHYTOCHROME INTERACTING FACTOR 4*), *CRY2* (*CRYPTOCHROMES 2*), *CO* (*CONSTANS*), and *FLC* (*FLOWERING LOCUS C*). (**E**) The rice OsFIP protein regulates the expression of *NTPase* and *threonine protease* genes through m^6^A modification. Abnormalities in this process lead to microspore degeneration. (**F**) MTA-mediated m^6^A modification affects the transcript stability and translation efficiency of key genes in the ABA (abscisic acid) signaling pathway, including *NCED5* (*9-cis-epoxycarotenoid dioxygenase 5*), *AREB1* (*ABA-responsive element-binding protein 1*) and *ABAR* (*ABA receptor*), thereby amplifying the ABA signal and promoting fruit ripening. Symbols: Upward arrow indicates increased gene expression; downward arrow indicates decreased gene expression.

Genetic evidence has unequivocally established the essential role of m^6^A writers in embryonic development [22,24,26]. In *Arabidopsis*, knockout mutants of core writer components *mta*, *mtb*, *vir*, and *fip37* arrest embryonic development at the globular stage, while knockdown mutants exhibit pronounced developmental defects, including shortened roots, increased branching, and aberrant vascular development [22,24,26] (Figure 2A). Thus, m^6^A modification is indispensable for normal embryogenesis and post-embryonic growth.

### 7.2. Vegetative Growth and Organ Development

#### 7.2.1. Shoot Apical Meristem Maintenance

The balance between stem cell proliferation and differentiation in the SAM is tightly regulated by m^6^A modification, as *fip37* mutants exhibit excessive shoot meristem proliferation [27]. FIP37-mediated m^6^A deposition on mRNAs encoding key SAM regulators, including *WUS* (*WUSCHEL*) and *STM* (*SHOOTMERISTEMLESS*), promotes their degradation, thereby maintaining the proper balance between stem cell self-renewal and differentiation [27]. Disruption of this m^6^A-mediated regulatory mechanism leads to meristem over-proliferation and developmental abnormalities [27] (Figure 2B).

#### 7.2.2. Trichome Development

m^6^A modification also influences epidermal cell differentiation [8,23,26]. *mta* knockdown lines exhibit increased trichome branching, a phenotype similar to that observed in *FIP37* overexpression plants [23,26]. Furthermore, the m^6^A reader ECT2 specifically regulates trichome morphology and branching by affecting the stability of mRNAs encoding trichome development-related genes [8].

#### 7.2.3. Organogenesis and Leaf Senescence

The m^6^A readers ECT2, ECT3, and ECT4 exhibit functional redundancy in controlling cell proliferation and organ morphology [80,81]. Arribas-Hernández et al. [80] first reported that *ect2*/*ect3* double mutants display significantly delayed leaf formation rates and aberrant leaf morphology. Subsequently, the same team further demonstrated that, in addition to leaf defects, *ect2*/*3* and *ect2*/*3*/*4* mutants exhibit slow root and stem growth, defective root gravitropism, abnormal flower and fruit morphology, and disrupted inflorescence phyllotaxis [80,81]. m^6^A writers are involved in leaf senescence, as MTA-deposited m^6^A counteracts dark-induced leaf senescence by promoting the decay of key senescence markers, including *SAG21* (*SENESCENCE-ASSOCIATED GENE 21*), *ORE1* (*ORESARA 1*), *NAP* (*NAC-LIKE, ACTIVATED BY AP3/PI*), and *NYE1* (*NON-YELLOWING 1*) [77] (Figure 2C).

### 7.3. Reproductive Development

#### 7.3.1. Floral Transition

m^6^A modification participates in the regulation of floral transition through multi-level molecular mechanism [29,39,82,83,84]. At the writers level, FIONA1 negatively regulates transcript levels of the blue light receptor *CRY2* (*CRYPTOCHROMES 2*), the light-responsive transcription factor *PIF4* (*PHYTOCHROME INTERACTING FACTOR 4*), and the positive flowering regulator *CO* (*CONSTANS*), while positively regulating *FLC* (*FLOWERING LOCUS C*) expression, thereby coordinately controlling *Arabidopsis* flowering time [29] (Figure 2D). OsYTH10 is involved in regulating flowering time in rice under long-day conditions by directly recognizing and promoting the degradation of flowering repressor mRNAs, leading to early flowering [82]. The m^6^A reader CPSF30-L plays a crucial role in floral transition by regulating APA site selection of flowering-related genes [39]. At the eraser level, GhALKBH5 efficiently erases m^6^A modifications from genes in the cotton photoperiodic flowering pathway, ensuring their stable expression and normal flowering [83]. ALKBH10B reduces m^6^A levels on transcripts of downstream flowering factors, including *FT* (*FLOWERING LOCUS T*), *SPL3* (*SQUAMOSA PROMOTER BINDING PROTEIN-LIKE 3*), and *SPL9* (*SQUAMOSA PROMOTER BINDING PROTEIN-LIKE 9*), through demethylation, thereby positively regulating their transcriptional abundance and ultimately promoting flowering in *Arabidopsis* [84].

#### 7.3.2. Reproductive Organ Development and Fruit Ripening

During reproductive organ development, *threonine protease* and *NTPase* gene expression is impaired in the rice *Osfip* mutant, controlling early microsporogenesis, and disruption of this regulation leads to microspore degeneration and male sterility [78] (Figure 2E). Fruit ripening involves complex m^6^A regulation [13,28]. In strawberry, the writer complex MTA/MTB deposits m^6^A on mRNAs of key ABA signaling pathway genes, including *NCED5* (*9-cis-epoxycarotenoid dioxygenase 5*), *AREB1* (*ABA-responsive element-binding protein 1*), and *ABAR* (*ABA receptor*), enhancing their mRNA stability or translation efficiency, thereby amplifying ABA signaling and accelerating fruit ripening [13] (Figure 2F). In tomato, the eraser SlALKBH2 interacts with the DNA demethylase SlDML2, catalyzing m^6^A demethylation and promoting SlDML2 expression, thereby accelerating ripening [28]. Conversely, SlDML2-mediated DNA methylation feedback-regulates SlALKBH2 transcription, co-regulating the fruit ripening process [28]. This study reveals a new layer of m^6^A regulation, achieving cross-level dialogue between the epitranscriptome and epigenome [28].

#### 7.3.3. Seed Germination

Seed germination is precisely regulated by ABA and gibberellic acid (GA) signaling, and m^6^A modification participates in this process through the coordinated action of erasers and readers [11,85,86]. The eraser ALKBH10B negatively regulates ABA signaling intensity by removing m^6^A modifications from ABA signaling factors, preventing its overactivation to ensure *Arabidopsis* normal germination [85]. In contrast, ALKBH6 exhibits an opposing function, as its loss leads to *Arabidopsis* germination insensitivity to ABA [86]. Furthermore, the m^6^A reader ECT1 promotes GA signaling by facilitating the degradation of *RGA1* (*REPRESSOR OF GA1-3 1*) mRNA and integrates *phyB* (*PHYTOCHROME B*) light signals, coupling endogenous hormone signals with environmental conditions to positively regulate *Arabidopsis* seed germination [11]. Collectively, m^6^A modification functions as a central regulator of seed germination by dynamically modulating the ABA/GA signaling balance and integrating environmental signals.

## 8. m^6^A as a Regulator of Abiotic Stress Responses

As sessile organisms, plants must continuously adjust their growth and development in response to environmental challenges. Extensive functional characterization has established m^6^A writers, readers, and erasers as key regulators of plant abiotic stress responses across multiple stress types and species (Table 2). Through coordinated modulation of mRNA stability, translation efficiency, ROS homeostasis, and hormone signaling pathways, these factors orchestrate stress adaptation (Table 2).

### 8.1. Drought and Water Deficit

#### 8.1.1. Regulation of Key Metabolic Pathways to Establish Stress Tolerance

**Figure 3:**
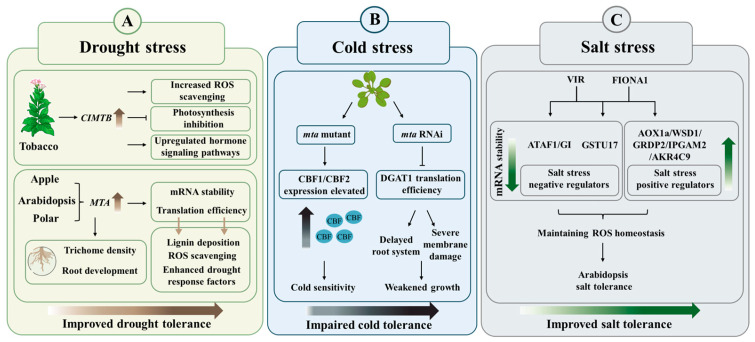
Regulatory mechanism model of plant abiotic stress tolerance mediated by m^6^A modification and its associated proteins. (**A**) Upregulating the expression of *MTB* (*homolog of human METTL14*) and *MTA* (*homolog of human METTL3*) enhanced the tolerance of plants to drought stress. In tobacco, upregulation of watermelon m^6^A methyltransferase *ClMTB* promotes ROS (reactive oxygen species) scavenging, activates hormone signaling pathways, and alleviates the inhibition of photosynthesis by water deficit. In apple, *Arabidopsis*, and poplar, upregulation of *MTA* improves the mRNA stability and translation efficiency of key genes, thereby promoting lignin deposition, enhancing ROS scavenging capacity and the function of drought-responsive factors, and regulating trichome density and root development under drought conditions. (**B**) Loss of *MTA* function (in *mta* mutants or RNAi lines) leads to impaired cold tolerance. In *mta* mutants, the expression of cold-responsive genes *CBF1*/*CBF2* (*C-repeat binding transcription factor 1*/*C-repeat binding transcription factor 2*) is abnormally elevated, increasing the sensitivity of *Arabidopsis* plants to low temperatures. In *mta* RNAi plants, the translation efficiency of *DGAT1* (*DIACYLGLYCEROL ACYLTRANSFERASE 1*) is reduced, causing delayed root development and severe cell membrane damage, resulting in growth retardation. (**C**) The m^6^A-associated proteins VIR (homolog of human VIRMA) and FIONA1 enhance *Arabidopsis* salt tolerance by finely modulating mRNA stability to maintain ROS homeostasis. VIR primarily functions by reducing the mRNA stability of negative regulators of salt stress such as *ATAF1* (*NAC transcription factor*), *GI* (*GIGANTEA*) and *GSTU17* (*glutathione S-transferase U17*). In contrast, FIONA1 increases the mRNA stability of positive regulators of salt stress such as *AOX1a* (*alternative oxidase 1a*), *WSD1* (*Wax ester synthase DGAT acyltransferase 1*), *GRDP2* (*glycine-rich domain-containing protein 2*), *IPGAM2* (*2-phosphoglycolate phosphatase 2*), *AKR4C9* (*aldo-keto reductase family 4 member C9*), etc. Symbols: In (**A**,**B**), the arrows indicate upregulation of gene expression. The T-shaped lines in (**A**,**B**) represent inhibition. In (**C**), the upward and downward arrows indicate an increase and decrease in mRNA stability, respectively.

Under drought stress, overexpression of the watermelon writers component *ClMTB* actively mobilizes genes involved in ROS scavenging, photosynthesis, and hormone signaling pathways, gradually establishing drought tolerance in tobacco [30] (Figure 3A). Similarly, in apple and *Arabidopsis*, MTA promotes the translation efficiency of genes involved in lignin biosynthesis, oxidative stress responses, and drought-responsive regulators under drought conditions to enhance drought tolerance [44,87] (Figure 3A). Recently, ECT10 was identified as an m^6^A reader that exerts a negative regulatory role by influencing the stability of both positive and negative regulators of the drought stress response [91].

#### 8.1.2. Influence on Epidermal Structure and Root Development to Enhance Drought Tolerance

Trichomes attached to plant epidermal tissues help plants effectively cope with abiotic stresses [96]. In poplar, plants overexpressing *PtrMTA* enhance adaptation to drought stress by increasing trichome density and promoting root growth and development [88,97] (Figure 3A).

#### 8.1.3. Regulation of Hormone Signaling to Modulate Drought Adaptive Responses

In tomato, SlALKBH9B has been shown to participate in drought stress responses, as *slalkbh9b* mutant plants exhibit induced ethylene production under drought conditions, accelerating flower abscission [97]. ALKBH10B, a well-studied eraser in plants, has been reported to function in recent studies [74,85,93,94,95]. Salt and drought stress-induced *ALKBH10B* expression negatively regulates physiological and biochemical processes, including photosynthesis, ROS homeostasis, and ABA responses, thereby reducing plant stress tolerance [74,85,93,94,95].

### 8.2. Temperature Stress

Under high-temperature conditions, m^6^A modification plays a crucial role in reproductive thermotolerance. AtALKBH10B enhances gene expression variability in floral organs, improving thermotolerance of reproductive structures and facilitating reproduction and survival in extreme environments [43]. Cold stress triggers dynamic m^6^A responses that enhance plant survival through regulation of stress-responsive transcripts [14,48]. Under low-temperature stress, Govindan et al. [48] subjected *Arabidopsis mta* mutants to 4 °C treatment for 3 h and 24 h and found that the expression levels of key cold tolerance transcription factors *CBF1* (*C-repeat binding transcription factor 1*) and *CBF2* (*C-repeat binding transcription factor 2*) were significantly altered, leading to increased cold sensitivity in the mutants (Figure 3B). Similarly, under 4 °C cold stress, Wang et al. [14] observed significantly weakened growth in *Arabidopsis MTA* RNAi plants, manifested as delayed root growth and more severe membrane damage (Figure 3B). Integrated analysis of the *Arabidopsis* m^6^A methylome, transcriptome and translatome revealed reduced translation efficiency of the cold stress response factor *DGAT1* without a decrease in transcript levels, suggesting positive regulation of translation by m^6^A marks [14]. In rice, the m^6^A reader ECT3 exhibits enhanced m^6^A-binding activity under cold stress due to reduced acetylation levels, leading to increased accumulation of cold-responsive gene transcripts and the establishment of cold tolerance in rice [89]. This post-translational regulation of reader activity adds another layer of control to the epitranscriptomic stress response [89].

### 8.3. Salt Stress

#### 8.3.1. Maintenance of Cellular Homeostasis Through Regulation of mRNA Stability

FIONA1, which is involved in photomorphogenesis and flowering in *Arabidopsis* [29], also plays a key regulatory role in the salt stress response [31]. Both VIR and FIONA1 enhance salt tolerance by maintaining ROS homeostasis, achieved through reducing the mRNA stability of *GSTU17* (*glutathione S-transferase U17*), a key negative regulator in the salt stress response [31,46] (Figure 3C). This pattern resembles that observed in drought and cold stress, where writers mediate tolerance by enhancing mRNA stability and translation efficiency [14,30,44,87]. Besides recognizing mRNA as a substrate, FIONA1 can also add methylation modifications to the spliceosomal U6 snRNA [29]. Therefore, the function of m^6^A extends beyond coding RNAs, directly influencing pre-mRNA splicing by regulating spliceosome function [25,29,98].

#### 8.3.2. Precise Regulation of Salt Stress Responses Through Opposing Mechanisms

Readers regulate salt responses through opposing mechanisms [10,46,90]. On the one hand, ECT8 and ECT12—the latter identified as a potential m^6^A reader—enhance the stability of positive stress-responsive gene transcripts by recognizing and binding their m^6^A modifications under salt and drought stress, thereby positively regulating plant stress resistance [10,90]. On the other hand, some readers promote the degradation of negative regulator mRNAs to relieve inhibition [47]. Specifically, the protein–protein interaction between ECT8 and DCP5 accelerates the degradation of salt stress-responsive factor mRNAs, actively clearing inhibitory factors to maintain physiological homeostasis under stress [47]. Thus, this reader-mediated degradation pathway selectively eliminates negative regulators, enabling effective stress responses. The regulatory function of erasers in salt stress is further exemplified by ALKBH8B. Overexpression of ALKBH8B in *Arabidopsis* enhances salt tolerance and reduces global m^6^A levels, accompanied by altered expression of both positive and negative salt stress regulators [92]. Moreover, ALKBH8B-overexpressing plants exhibit enhanced cotyledon greening upon ABA treatment, with decreased transcript levels of ABA signaling-related genes *ABI* and *ABI4*, suggesting that ALKBH8B integrates salt stress and ABA signaling pathways through m^6^A demethylation [92].

**Figure 4:**
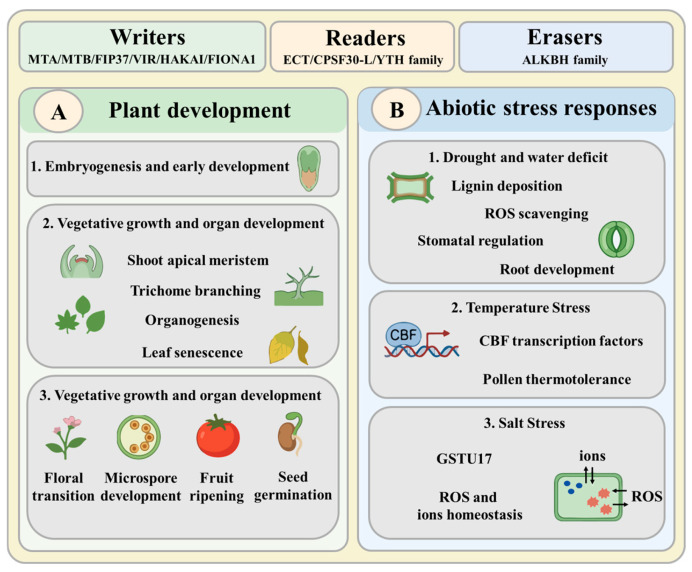
Overview of m^6^A regulatory factors (writers/methyltransferases, readers/m^6^A-binding proteins, and erasers/demethylases) and their functions in plant development and abiotic stress responses. (**A**) Roles of m^6^A regulators in plant development, including embryogenesis and early development, vegetative growth and organogenesis (shoot apical meristem, trichome branching, organogenesis, and leaf senescence), and reproductive development (floral transition, microspore development, fruit ripening, and seed germination). (**B**) Roles of m^6^A regulators in abiotic stress responses, including drought and water deficit (lignin deposition, ROS/reactive oxygen species scavenging, stomatal regulation, and root development), temperature stress (*CBF*/*C-repeat binding transcription factor*) and salt stress (*GSTU17*/*glutathione S-transferase U17*, ROS, and ion homeostasis).

The m^6^A modification has emerged as a central epitranscriptional regulator integrating plant development with abiotic stress responses (Figure 4). Writers deposit methylation marks interpreted by readers to control mRNA stability, translation, and alternative polyadenylation, while erasers confer reversibility for rapid stress reconfiguration. This mechanistic versatility allows a single chemical mark to orchestrate diverse processes from embryogenesis to stress tolerance.

## 9. Perspectives

Despite significant progress, many questions remain. The molecular machinery linking m^6^A to translation initiation in plants is unclear. The upstream signals controlling writer and eraser activities require characterization. The specific readers mediating crosstalk between m^6^A and chromatin modifications have yet to be identified. And the functional significance of individual m^6^A sites remains largely unknown.

Looking forward, manipulating RNA modifications represents a new frontier in crop improvement. Developing single-base editing tools using CRISPR/dCas13 systems fused with writer or eraser domains will enable precise, locus-specific manipulation of m^6^A. Integrating single-cell m^6^A sequencing, multi-omics, and advanced gene-editing will reveal the dynamic and functional specificity of m^6^A modifications. Because m^6^A converges on common stress signaling hubs (e.g., ROS and ABA), epitranscriptome engineering holds promise for enhancing tolerance to combined stresses in the field. Furthermore, natural variation in m^6^A regulators and targeted editing of cultivar-specific m^6^A sites may accelerate breeding of stress-resilient, high-yielding crops. As climate change intensifies agricultural challenges, epitranscriptome engineering may become a transformative strategy for developing stress-resilient crops.

## Figures and Tables

**Table 1:** The role of confirmed m^6^A writers, readers and erasers in plant development.

Type	Name	Plant Species	Function	References
Writers	MTA	*Arabidopsis*	Trichome branching and leaf senescence	[23,77]
	MTA/MTB	Strawberry	Fruit ripening	[13]
	MTA/MTB/FIP37	*Arabidopsis*	Embryonic development	[22,24]
	FIP37	*Arabidopsis*	Stem cell proliferation and differentiation	[27]
	FIP	Rice	Microspore development	[78]
	HAKAI/VIR	*Arabidopsis*	Embryonic development	[24]
	FIONA1	*Arabidopsis*	Flower transformation and photomorphogenesis	[25,29,79]
Readers	ECT1	Tomato	Seed germination	[11]
	ECT2/3/4	*Arabidopsis*	Leaf morphology Cell proliferation	[80,81]
	CPSF30-L	*Arabidopsis*	Nitrate signaling Floral transition ABA signaling responses	[38,39]
	YTH10	Rice	Promoting early flowering	[82]
Erasers	ALKBH2	Tomato	Fruit ripening	[28]
	ALKBH5	Cotton	Affecting gene expression stability and transcriptional levels	[83]
	ALKBH9B	*Arabidopsis*	Regulating viral infection	[42]
	ALKBH10B	*Arabidopsis*	Floral transition and seed germination	[84,85]

Writers: Methyltransferases; Erasers: Demethylases; Readers: m^6^A-binding proteins; MTA: Homolog of human METTL3; MTB: Homolog of human METTL14; FIP/FIP37: Homolog of human WTAP; VIR: Homolog of human VIRMA; HAKAI: a ubiquitin E3 ligase; ECT: EVOLUTIONARILY CONSERVED C-TERMINAL REGION; CPSF30-L: CLEAVAGE AND POLYADENYLATION SPECIFICITY FACTOR 30; YTH: YT521-B homology; ABA: Abscisic acid; ALKBH: the a-ketoglutarate-dependent dioxygenase homolog.

**Table 2:** The role of confirmed m^6^A writers, readers and erasers in abiotic stress.

Type	Name	Plant Species	Abiotic Stress Type	Function	References
Writers	MTA	Apple	Drought	Lignin synthesis and oxidative stress response	[44]
	MTA	*Arabidopsis*	Drought	Affecting mRNA stability and translation efficiency	[87]
	MTA	Poplar	Drought	Trichome and root development	[88]
	MTA	*Arabidopsis*	Low temperature	Affecting mRNA stability	[14,48]
	MTB	Tobacco	Drought	ROS scavenging Photosynthesis Hormone signaling	[30]
	FIONA1/VIR	*Arabidopsis*	Salt	Maintaining ROS homeostasis	[31,46]
Readers	ECT3	Rice	Cold	mRNA accumulation	[89]
	ECT8	*Arabidopsis*	Salt	mRNA degradation Affecting mRNA stability	[47,90]
	ECT10	*Arabidopsis*	Drought	Affecting mRNA stability	[91]
	ECT12	*Arabidopsis*	Drought/Salt	Affecting mRNA stability	[10]
Erasers	ALKBH6	*Arabidopsis*	Abiotic stress and hormones	Seed germination and seedling growth	[86]
	ALKBH8B	*Arabidopsis*	Salt and hormones	Salt stress response and ABA signaling response	[92]
	ALKBH9B	Tomato	Drought	Regulating ethylene levels to suppress drought-induced abscission	[79]
	ALKBH10	Cotton	Salt	Disruption of ROS homeostasis and ion homeostasis	[93]
	ALKBH10B	Cotton	Drought	Negative regulation of photosynthesis and ABA signaling	[94]
	ALKBH10B	Cotton	Drought	Affecting ABA and Ca^2+^ signaling transduction pathways	[74]
	ALKBH10B	Tomato	Drought/Salt	Photosynthesis weakens, proline accumulation decreases, ROS levels rise, and cellular damage intensifies	[95]
	ALKBH10B	*Arabidopsis*	Heat	Enhanced gene expression variability	[43]
	ALKBH10B	Potato	Salt	Affecting mRNA stability and translation efficiency	[75]

Writers: Methyltransferases; Erasers: Demethylases; Readers: m^6^A-binding proteins; MTA: Homolog of human METTL3; MTB: Homolog of human METTL14; FIP37: Homolog of human WTAP; FIONA/VIR: Homolog of human VIRMA; ROS: Reactive oxygen species; ECT: EVOLUTIONARILY CONSERVED C-TERMINAL REGION; ABA: Abscisic acid; ALKBH: the a-ketoglutarate-dependent dioxygenase homolog.

## Data Availability

No new data were created or analyzed in this study.
